# Erratum to “Performance of gender detection tools: a comparative study of name-to-gender inference services,” 2021;109(3):414–21 and “Using genderize.io to infer the gender of first names: how to improve the accuracy of the inference,” 2021;109(4):609–12.

**DOI:** 10.5195/jmla.2022.1528

**Published:** 2022-04-01

**Authors:** Paul Sebo

The reference for Gender API in both of these manuscripts linked to the wrong Gender API tool. The correct URL is <https://gender-api.com/en/>. The original articles have both been updated to reflect this change.

